# Integrative Omics Analysis Reveals Metabolic Features of Ground-Glass Opacity-Associated Lung Cancer

**DOI:** 10.7150/jca.92437

**Published:** 2024-02-04

**Authors:** Shuai Shi, Dayuan Luo, Yanyi Yang, Xiang Wang

**Affiliations:** 1 Department of Thoracic Surgery, Second Xiangya Hospital, Central South University, 410011, Changsha, Hunan Province, China.; 2 Heath Management Center, Second Xiangya Hospital, Central South University, 410011, Changsha, Hunan Province, China.

**Keywords:** ground-glass opacity, metabolomics, lung neoplasms, transcriptome, single-cell RNA sequencing

## Abstract

**Background:** Ground-glass opacity (GGO)-associated cancers are increasingly prevalent, exhibiting unique clinical and molecular features that suggest the need for a distinct treatment strategy. However, the metabolic characteristics and vulnerabilities of GGO-associated lung cancers remain unexplored.

**Methods:** We conducted metabolomic and transcriptomic analyses on 40 pairs of GGO-associated lung cancer tissues and adjacent normal tissues. By integrating data from TCGA database and single-cell RNA sequencing, we aimed to identify aberrant metabolic pathways, establish a metabolite-associated gene signature, and pinpoint key metabolic genes. The physiological effect of key genes was detected in vitro and vivo assays.

**Results:** We identified a 30-gene metabolite-associated signature and discovered aberrant metabolic pathways for GGO-associated lung cancer at both metabolic and transcriptional levels. Patients with this signature displayed specific prognostic and molecular features. Cox regression analysis, based on the Cancer Genome Atlas Program (TCGA) data, further narrowed down the metabolite-related gene signature, resulting in a 5-gene signature. Confirmed by single-cell RNA sequencing (GSE203360), the 5-gene signature was mainly expressed in cancer cells of GGO tissue. Real-time quantitative PCR (RT-qPCR) further validated the differential expression of these genes between GGO and adjacent normal tissue obtained from pulmonary surgery. Finally, our integrative analysis unveiled aberrant histidine metabolism at both the multi-omics and single-cell levels. Moreover, we identified MAOB as a key metabolic gene, demonstrating its ability to suppress cell proliferation, migration, and invasion in LUAD cell lines, both in vitro and in vivo.

**Conclusions:** We identified a specific metabolite-associated gene signature and identified aberrant histidine metabolism in GGO-associated lung cancer from multiple perspectives. Notably, MAOB, a crucial component in histidine metabolism, demonstrated a significant inhibitory effect on the proliferation and metastasis of LUAD, indicating its potential significance in pathogenesis and therapeutic interventions.

## Introduction

Ground-glass opacity (GGO) is characterized by an irregular or round-like shadow with increased density in computed tomography (CT) images. The prevalence of low-dose CT for lung cancer screening has led to an increased detection of non-solid pulmonary nodules containing GGO components 1. The Early Lung Cancer Action Program (ELCAP) reported a GGO detection rate of 4.4% and approximately 20% of lung adenocarcinoma (LUAD) manifested as GGO 2. Moreover, a multi-center study from a Chinese hospital revealed 95.5% of lung cancer patients presented as GGO, calling for further attention to GGO-associated lung cancer 3.

GGO-associated lung cancers exhibit unique clinical and molecular features in comparison to solid-nodule lung cancers. Typically, GGO-associated lung cancers are generally lung adenocarcinoma and relatively indolent, characterized by a non-invasive pathology type and a favorable prognosis 4, 5. Segmentectomy, rather than lobectomy, has been shown to achieve nearly 100% 5-year relapse-free survival for GGO patients 6. Numerous studies have uncovered unique genetic and immune features of GGO-associated lung cancers, indicating a less active immune microenvironment and lower tumor mutant burden than solid-nodule lung cancers 7-9. However, the metabolic characteristics of GGO-associated lung cancer are not well-understood. Additionally, existing treatment strategies are primarily based on clinical features, lacking an understanding of these unique molecular characteristics.

Metabolic reprogramming has emerged as a hallmark of cancer 10. Cancer cells adaptively alter their metabolism, including glycolysis, lipid metabolism, to sustain growth in a nutrient-deficient environment 11-13. In the context of lung cancer, metabolomic was initially employed to detect differential metabolites in serum and urine for disguising healthy individuals and those with cancer 13, 14. However, these studies sorely reflected the altered metabolic phenomena, rather than underlying mechanism of carcinogenesis and progression. The integration of metabolomic and transcriptomics could reveal consistent metabolic alterations at both the metabolites and mRNA levels, providing insight into molecular mechanisms and targeted therapies of lung cancer. Current integrative studies have identified metabolite-related gene signatures and key metabolic pathways aberrantly regulated in lung adenocarcinoma and squamous cell carcinoma 15. Nevertheless, none of these studies have focused on GGO-associated lung cancer.

In this study, we integrated metabolic profile and gene expression of GGO-associated lung cancer through metabolomic and transcriptomic analyses, to identify metabolite-associated gene signature and aberrant metabolic pathway. The metabolite-associated genes were further refined by assessing their prognostic and physiological significance using data from TCGA database and single-cell RNA sequencing, to identify metabolic genes crucial for carcinogenesis and progression of lung cancer. Ultimately, we demonstrated aberrant histidine metabolism at both multi-omics integrative analysis and single-cell level, and identified the critical role of MAOB in LUAD carcinogenesis and progression, substantiated through vitro and vivo assays.

## Materials and Methods

### Biological sample collection

We conducted assessments on patients who underwent pulmonary resection surgery at the Second Xiangya Hospital from March 2019 to June 2020. The inclusive consisted:1) preoperative CT confirmed pulmonary lesions as GGO, which refers to a homogeneous increase in density in the lung field that does not completely obscure the bronchiolovascular structures 16; 2) intraoperative biopsy confirming LUAD as the pathology of the target lesion. Exclusion criteria included: 1) multiple primary pulmonary nodules with malignancy potential; 2) preoperative examinations indicating positive lymph nodal or distant metastases; 3) patients with a history of malignancy. Additionally, we specifically selected GGO-associated nodules within a10mm diameter, indicative of the early stage of lung cancer. Each eligible patient provided a pair of specimens from resected lesions, including GGO and adjacent normal tissue. Eventually, 40 pairs of samples were collected for subsequent analysis, with 15 pairs used for metabolomic analysis and 25 pairs used for transcriptomics analysis. The fresh specimens underwent a process including washing by saline, transfer into clean tubes, and rapid freezing in liquid nitrogen until analysis.

### Untargeted Metabolomics analysis of GGO

An Untargeted metabolomics, aiming to capture all measurable mass spectral signals, was performed by XploreMET platform (Metabo-profile, Shanghai, China). Each sample was assigned a specific barcode for identification and logged into an administration system for traceability. The metabolites extraction, purification, and derivatization were performed in a streamlines processing. Chromatographic peaks of metabolites were measured using a time-of-flight mass spectrometer with gas chromatography (GC-TOF/MS). Peak filtering was performed to eliminate interfering signals, and the remaining signals were matched with a standard library (JiaLib ^TM^) to specific metabolites identification. Missing value was replaced with half of the minimum positive value and raw data underwent normalization through log transformation and auto scaling.

### Identification and pathway enrichment of differentially expressed metabolites

The orthogonal partial least squares-discriminant analysis (OPLS-DA) was performed to identify differentially expressed metabolites (DEMs). Metabolites with variable importance in projection (VIP) value greater than 1.0 were considered significant in the OPLS-DA model. Overfitting effect was assessed through permutation testing and a well-behaved model is favored by the results that R2 and Q2 values exceeded 0.5. Significance analysis of microarrays (SAM) was conducted, and DEMs with a false discovery rate (FDR) value less than 0.01were selected. Additionally, a volcano plot, using t-tests and fold change analysis, was generated, with statistical significance defined as a fold change (FC) exceeding 1.25X and an FDR value less than 0.25. DEMs were finally identified as the common metabolites in the three algorithms above. Quantitative enrichment analysis was performed to reflect the metabolic alteration based on the DEMs. All the algorithms above were performed by MetaboAnalyst 5.0.

### Transcriptomics analysis of GGO

25 pairs of samples underwent high-throughput transcriptome sequencing using BBGISEQ platform (BGI Tech, Beijing, China). Expression profiling was quantified in each sample and compared against a reference genome deriving from NCBI (version. GCF_000001405.38_GRCh38.p12). The Wilcoxon signed-rank test was conducted to select the differentially expressed genes (DEGs) between GGO and paired normal tissue. A volcano plot was generated with a threshold of log_2_(FC) exceeding 0.75X and P value less than 0.01. Principal component analysis (PCA) was utilized to confirm the data clustering. All these analyses were conducted using R studio.

### Integrated metabolomics and transcriptomics analysis

We determined genes associated with DEMs using the Human Metabolome Database (HMDB, RRID:SCR_007712). A multi-omics gene signature was identified as the overlapped part between metabolites-associated genes (MAGs) and DEGs. A Kyoto Encyclopedia of Genes and Genomes (KEGG) enrichment analysis of MAGs was performed by Omicshare. By using MetaboAnalyst and Cytoscape (Version 3.9.1), joint network analysis including DEMs and MAGs was performed and revealed the alteration in metabolomics and transcriptomics, synchronously.

### Identification of multi-omics associated subtypes based on TCGA data

We utilized R-package “ConsensusClusterPlus” to identify the subtypes based on the multi-omics gene signature. K-means consensus clustering algorithm was iterated multiple times to enhanfe clustering stability. The scores of tumor-associated metabolic physiological pathways were calculated in Cancer Genome Atlas Program (TCGA) LUAD samples of two different subtypes, by using ssGESA algorithm in R-package “GSVA”. Survival association between different subtypes was assessed by using R-package “survminer” and the log-rank test.

### Screening for metabolite-associated genes with prognostic value

For further screening for metabolite-associated genes with prognostic value, we initially conducted a univariate analysis, considering significance at P value < 0.05. The identified candidate genes then underwent multivariate cox regression analysis. Metabolite-related risk genes were determined using a stepwise regression method, selecting genes when the Akaike information criterion (AIC) value reached its minimum 17. The risk score of TCGA sample was calculated based on gene expression, and Kaplan-Meier analysis was performed between high and low-risk groups by using R-package “survminer”. The receiver operating characteristic (ROC) curve of risk type and clinical features was generated using R package 'timeROC'. A Nomogram, including independent prognostic factors, was constructed using the R package “RMS”. A heatmap displaying the distribution of clinical features and multi-omics subtypes in different risk groups was created using R package “ComplexHeatmap”.

### Single-cell RNA sequencing reveals the distribution of metabolite-associated gene signature at the cellular level

We downloaded GGO Sc-RNA sequencing data from GSE203360. The data processing, including filtering, normalization and selection of highly variable genes, was performed by R package “Seurat”. Then, uniform manifold approximation and projection analysis (UMAP) was applied for dimension reduction and clustering. The 'FindAllMarkers' function was utilized to identify markers between different UMAP clusters, with the criteria set as | Log_2_FC | value < 0.25, P-adjusted value < 0.05 and 'min.pct' equal to 0.3. Based on differentially expressed genes, cell clusters were annotated based on R package “SingleR” and gene markers from the CellMaker database. The R package “AUCell” was employed to select metabolite-associated gene signature enriched cells. GSEA analysis was processed to reveal the alternative pathways of the metabolite-associated gene signature enriched cells.

### Quantitative Real-time PCR (RT-qPCR)

Fresh GGO tissue obtained from pulmonary resection surgery underwent total RNA extraction using RNAiso (Takara, 9109). The reverse transcription of total RNA was performed by cDNA Synthesis SuperMix Kit (Yeasen, 11141ES60). The real-time quantitative PCR (RT-qPCR) experiment was executed by SYBR Green Master Mix Kit reagents (Yeasen, 11203ES08) on a StepOnePlus instrument. The methods for using these reagents were consistent with the instructions provided by the manufacturers. The primers of detected genes for RT-qPCR are listed in Table 1.

### Collection, analysis and processing of data from public databases

Kaplan-Meier curves of the 5-gene signature were generated using “lung cancer” module of Kaplan-Meier Plotter, which includes data from TCGA and GEO databases. The gene list of histidine metabolism was downloaded from the KEGG database. Protein-protein interaction networks for histidine metabolism was constructed using STRING database and Cytoscape (Version 3.9.1). Protein expression of MAOB in clinical samples was acquired from proteomics module of UALCAN. Immunohistochemistry stain of lung cancer sample was acquired from The Human Protein Atlas, and the following criteria were applied: 0 (no staining), 3 (weak staining), 6 (moderate staining), and 9 (strong staining).

### Cell culture and antibodies

293FT cells, normal human bronchial epithelial cells (16HBE) and human LUAD cells (A549, H1299, PC9 and H1975) were provided by the Cancer Research Institution of Central South University. Short tandem repeat analysis verified the viability of these cells prior to the experiment and cells were cultured in RPMI 1640 (Gibco) and DMEM (Gibco) medium with 10% fetal bovine serum (FBS) (164210-50, Procell), 1% penicillin-streptomycin (C100C5, NCM).

### The establishment of MAOB overexpression cell lines

The coding sequence of MAOB was cloned into Ubi-MCS-3FLAG-CBh-gcGFP-IRES-puromycin and packaged as lentiviruses in 293FT cells (Genechem, Shanghai, China). The target and corresponding vector virus were transfected into A549 and PC9 cell lines and were removed at 24 h after transfection. Puromycin was employed to select positive cells using a concentration series, and the efficacy of transfection was assessed by western blot assay.

### Immunohistochemistry (IHC)

We also collected GGO samples and tumor tissues from nude mice for IHC. Tissue slides underwent deparaffinization, rehydration through turpentine and alcohol series, followed by antigen retrieval using sodium citrate buffer. The slides were treated with 3% H_2_O_2_ and blocked with 5% goat serum for 30 min at room temperature. GGO sections were then incubated by primary antibodies MAOB (12602-1-AP, Proteintech), Ki67 (28074-1-AP, Proteintech), PCNA (60097-1-Ig, Proteintech), Vimentin (10366-1-AP, Proteintech), E-cadherin (20874-1-AP, Proteintech) and N-cadherin (22018-1-AP, Proteintech) overnight at 4°C and secondary antibody 30 min at 37°C. The final stain was performed by using DAB.

### Western blot assay

Cells in a 60-mm^2^ were lysed in 200 µL strong RIPA buffer (P0013B, Beyotime), which contained protease inhibitor cocktail (20124ES03, Yeasen). The protein concentration was determined using the BCA protein assay kit (P0011, Beyotime). Proteins were separated on a 4%-20% preformed gel (ET12420Gel, ACE) and transferred to PVDF membranes (Millipore). The membranes were blocked with 5% skim milk in TBST at room temperature for 2h, followed by incubation with the MAOB primary antibody (1:2000) (12602-1-AP, Proteintech) at 4°C overnight. After washing 3 times with TBST, the membrane was incubated with the secondary antibody (1:8000) (SA00001-2, Proteintech). Finally, the target proteins were visualized using a hypersensitive ECL kit (P0018S, Beyotime).

### Cell proliferation experiments

Cell proliferation ability was assessed by CCK-8 and colony formation assays. For the CCK-8 assay, 2000 cells were cultured in each well of 96-well plate, and the cell counting kit-8 regent (C0005, Topscience) was added at the appropriate time points following the instructions. The OD 450 values of each well were measured 2h after the addition. For the colony formation assay, 1000 cells were plated in each well of 6-well plate and cultured for 10 days. Then the cells were fixed with 4% paraformaldehyde and stained with 0.2% crystal violet for imaging.

### Migration and invasion assays in a transwell system

The transwell system with 8.0-μm pores (Corning) was employed to measure the migration and invasion ability of detected cells. For assessing migration ability, 1×10^4^ PC9 cells per well or 2×10^4^ A549 cells per well in 150μL 1640 medium without FBS were placed in the upper chamber, and 600µL 1640 medium with 10% FBS was added to the bottom chamber. In addition, Matigel (3556234, Corning) was added to the upper chamber in advance for detecting cell invasion according to the reagent instructions. After 24 h of culture, cells on the upper chamber were washed by PBS, fixed with ethanol and stained with 0.2% crystal violet.

### Nude mice and study approval

A total of 5 × 10^6^ cells with MAOB overexpression were injected subcutaneously into 5-week-old female nude mice (SJA Laboratory Animal, Changsha China). When the tumors reached the maximum diameter according to ethical criteria of our institution, all the mice were humanely euthanized by cervical dislocation, and the tumors were weighed after removal. All experimental procedures for animal study were approved by the Institutional Animal Care and Use Committee of Central South University and strictly complied with the legal mandates and national guidelines for the care and maintenance of laboratory animals.

## Results

### Study population and sample collection

From March 2019 to June 2020, samples from 40 patients who underwent pulmonary resection surgery in second Xiangya Hospital were analyzed through metabolomics and transcriptomic. Each patient was identified as having a GGO component by preoperative CT scans and confirmed with lung cancer through postoperative pathology. Of note, all GGO-associated lung cancers in this study were diagnosed as lung adenocarcinoma (LUAD). 15 out of 40 samples were subjected to metabolomics while 25 samples were subjected to transcriptomics using simple randomization. The clinical and pathological characteristics of the study population showed no differences between metabolomic group and transcriptomic group (Table 2). Among the 40 patients, 24 had pure GGO nodules, and 16 had mixed GGO nodules, consisting of GGO and a solid component. GGO nodules were relevant to the early stage of lung cancer in pathology, with 22 patients having in situ and minimally invasive LUAD, while only 18 patients had invasive LUAD.

### Metabolomic fingerprinting reveals significant metabolites and aberrant metabolic pathways in GGO-associated lung cancer

**Figure 1:**
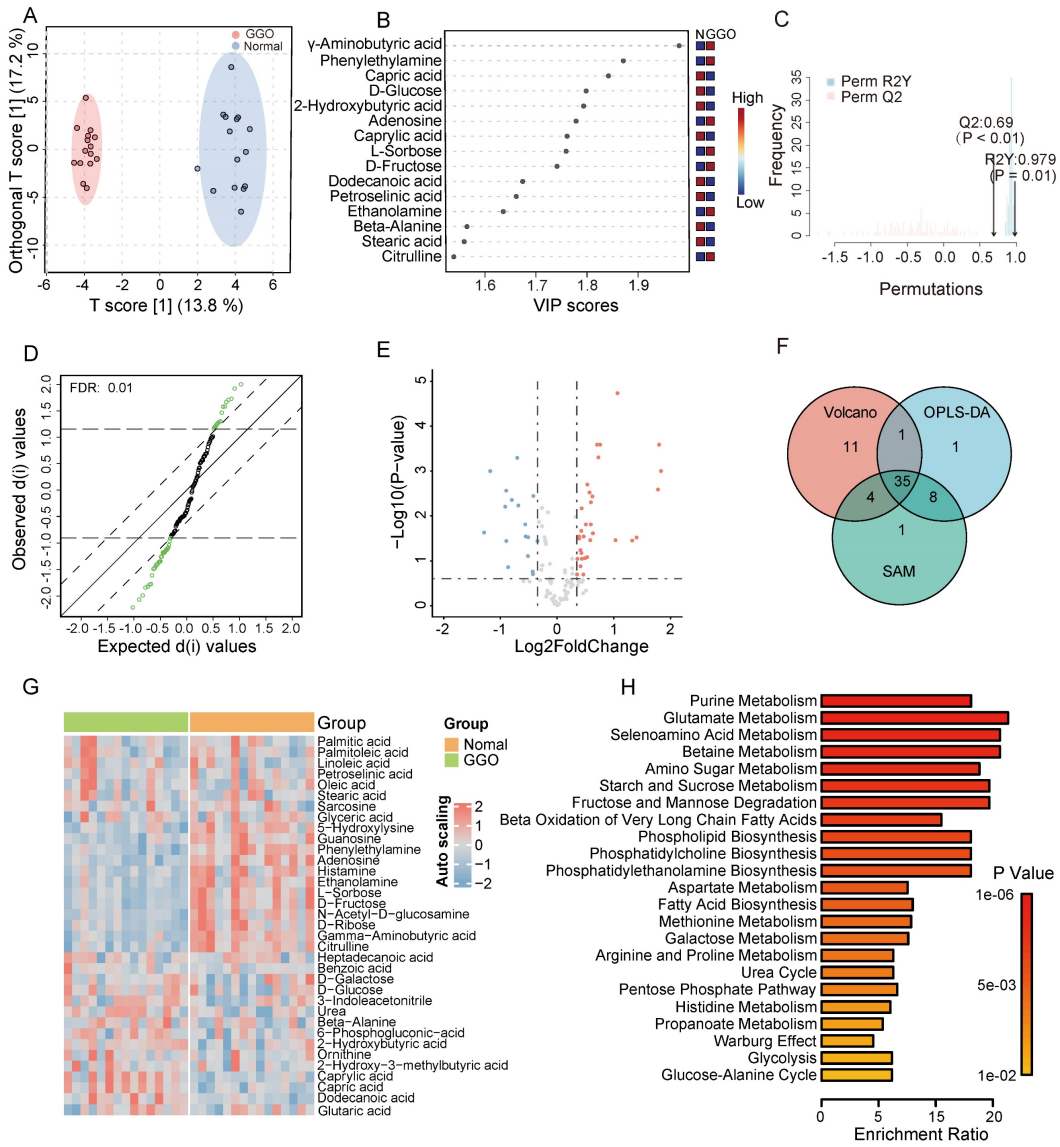
** Metabolomics analysis of GGO-associated lung cancer and adjacent normal tissue. A.** The scores plot from Orthogonal Partial Least Squares-Discriminant Analysis (OPLS-DA) distinctly demonstrated a clustering effect between GGO tissue and normal tissue. **B.** The Variable important in projection (VIP) scores suggested significant metabolites in the OPLS-DA model for distinguishing GGO and normal tissues. **C.** The permutation test demonstrates the OPLS-DA model had no overfitting effect, with reliable Q2 value (0.69) and R2Y value (0.979). **D.** Significance Analysis of Microarrays (SAM) suggested significant metabolites for distinguishing GGO and normal tissue. **E.** Volcano plot demonstrated significant metabolites distinguishing GGO and normal tissues (FDR<0.25, fold change exceeding 1.25X). **F.** The venn diagram of OPLS-DA, SAM and volcano revealed 35 underlying metabolites for distinguishing GGO and normal tissues. **G.** Heatmap of 35 significant metabolites between GGO and normal tissues. **H.** Quantitative enrichment analysis of 35 significant metabolites.

According to GC-TOF/MS analysis, we detected and annotated 121 metabolites in GGO and adjacent normal tissue. To identify metabolic alterations in GGO, we performed OPLS-DA analysis, and score plots demonstrated a clear cluster between GGO and adjacent normal tissues (Figure 1A). VIP score reflected the contribution of each metabolite to the discrimination model, and we selected 45 metabolites with VIP value greater than 1.0 as significant biomarkers (Figure 1B). The permutation test, with a Q2 value 0.69 and a R2 value 0.797, demonstrated that the OPLS-DA model had no overfitting effect (Figure 1C). For further screening significant metabolites in GGO, we performed significance analysis of microarrays (SAM) and selected 48 metabolites (Figure 1D). Based on fold change analysis (fold change exceeding 1.25X) and T-tests (FDR < 0.25), we identified 34 significant upregulated metabolites and 17 significant downregulated metabolites in GGO tissue compared to adjacent normal tissues (Figure 1E). Ultimately, Venn's diagram showed 35 metabolites were common in OPLS-DA, SAM and volcano plot (Figure 1F), identifying them as differentially expressed metabolites (DEMs). A heatmap illustrated the relative expression of DEMs in GGO and adjacent normal tissue (Figure 1G). Subsequently, we performed a quantitative enrichment analysis on DEMs to explore potential metabolic alterations in the GGO group. The top 5 of enriched pathways were purine metabolism, glutamate metabolism, selenoamino acid metabolism, betaine metabolism and amino sugar metabolism (Figure 1H).

### Transcriptomics analysis of GGO

We performed a differentially expressed genes (DEGs) analysis based on high-throughput sequencing in samples of transcriptional group. With a threshold log_2_(FC) exceeding 0.75X and P value less than 0.01, we identified 1380 DEGs between GGO and adjacent normal tissue, with 532 DEGs up-regulated and 848 DEGs down-regulated (Figure S1A and B). PCA analysis showed a clear separation between samples of different groups (Figure S1C).

### Integrative analyses of metabolomics and transcriptomics identify metabolite-associated gene signature and aberrant metabolic pathways in GGO-associated lung cancer

**Figure 2:**
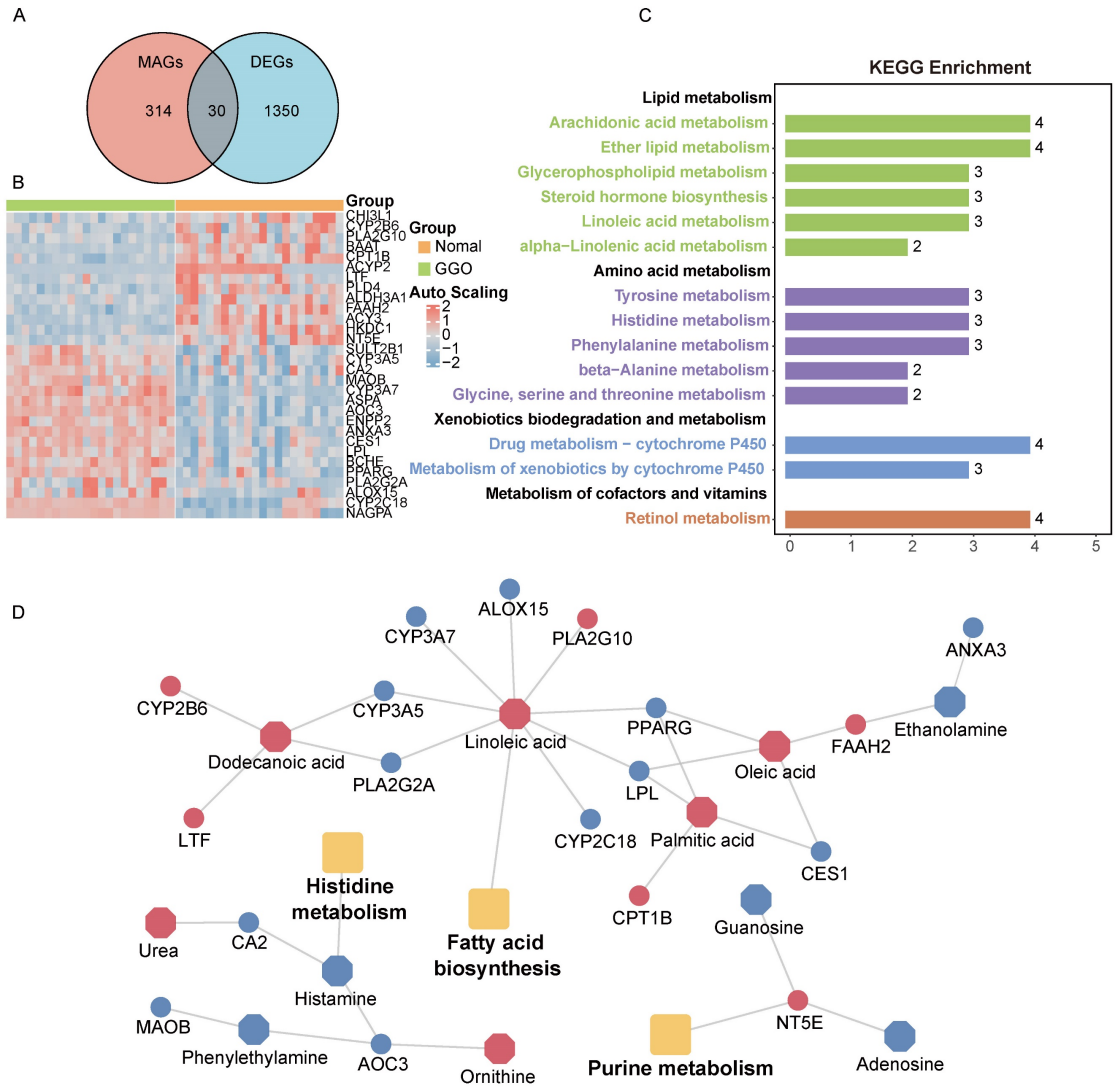
** Integrative analyses of metabolomics and transcriptomics in GGO-associated lung cancer. A.** Venn diagram of metabolite-associated genes (MAGs) and differentially expressed genes (DEGs), identified a 30-gene multi-omics signature. **B.** Heatmap of the 30-metabolite-gene signature between GGO and normal tissues.** C.** Metabolism-associated KEGG pathway analysis of the 30-metabolic-gene signature. **D.** Joint network analysis aberrant metabolic pathways both at the metabolic and transcriptional levels.

Metabolites represent downstream products of gene expression, and the intensity of metabolites can be influenced by multiple factors. To explore primary metabolic changes in GGO associated lung cancer, we integrated metabolomics and transcriptomics to screen coincident variations. We mapped the DEMs to their corresponding metabolic genes using human metabolome database (HMDB), resulting in 343 candidate metabolite-associated genes (MAGs). The integration of candidate MAGs and DEGs identified 30 common genes (Figure 2A). The heatmap of the 30-gene signature displayed the expression level in GGO and adjacent normal tissue (Figure 2B). We performed metabolism-associated KEGG pathway enrichment analysis on the 30-gene signature and most of the enriched metabolic pathways were related to lipid metabolism and amino acid metabolism (Figure 2C). To further explore altered metabolic progression both in metabolomics and transcriptomic, we subjected 35 DEMs and 30 MAGs to joint network analysis. The result suggested aberrant histidine metabolism, fatty acid biosynthesis and purine metabolism both in metabolic and transcriptional levels (Figure 2D).

### Clusters based on metabolite-associated gene signature have distinct prognostic and physiological features

**Figure 3:**
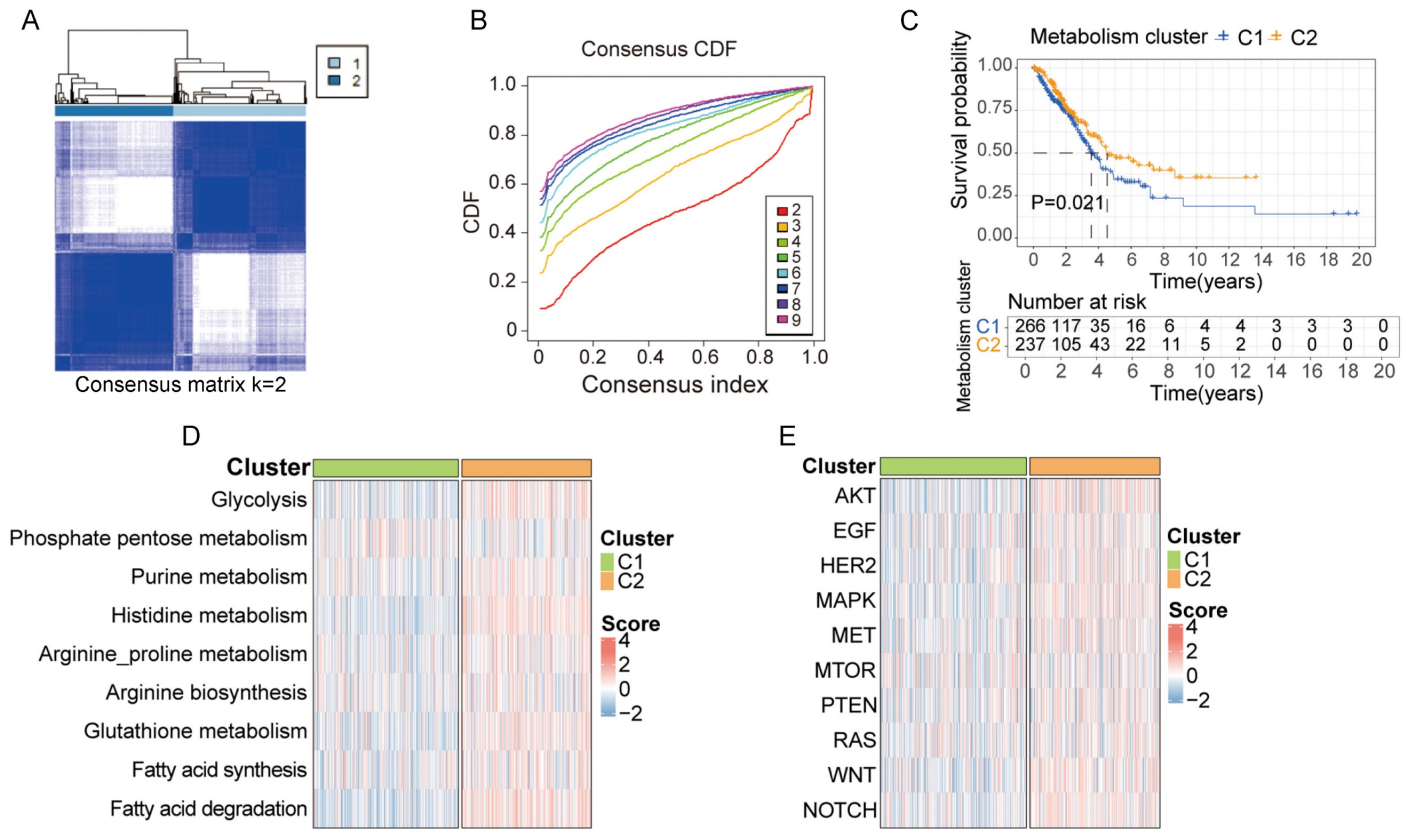
** Clusters based on metabolite-associated gene signature have distinct prognostic and physiological features. A, B.** Using the k-means clustering algorithm, 503 lung cancer samples from TCGA were divided into 2 clusters based on 30-metabolic-gene signature when k value was set to 2. **C.** Patients in cluster 1 and cluster 2 exhibited different prognostic features. **D, E.** Patients in cluster 1 and cluster 2 exhibited distinct metabolic and physiological features.

Based on the 30-gene multi-omics signature, we conducted k-means consensus clustering analysis on 503 LUAD samples from TCGA. When the k value was set to 2, the TCGA samples were divided into 2 subgroups (C1 and C2), demonstrating a favorable clustering effect (Figure 3A and B). We observed significant differences in prognosis between patients in cluster C1 and C2 (Figure 3C). Additionally, we calculated the score of each sample based on common metabolic and oncogenic pathways using ssGESA algorithm (Figure 3D and E). Samples in cluster C2 exhibited higher expression in multiple tumor-associated metabolic pathways, including glycolysis, purine metabolism, histidine metabolism, arginine and proline metabolism, arginine biosynthesis, glutathione metabolism, fatty acid synthesis and degradation (Figure S2A). Similarly, samples in cluster C2 displayed higher expression in multiple tumor-associated physiological pathways including AKT, EGF, HER2, MAPK, MET, mTOR, PTEN, RAS, WNT and NOTCH signal pathway (Figure S2B).

### Screening for metabolic genes with prognostic value

**Figure 4:**
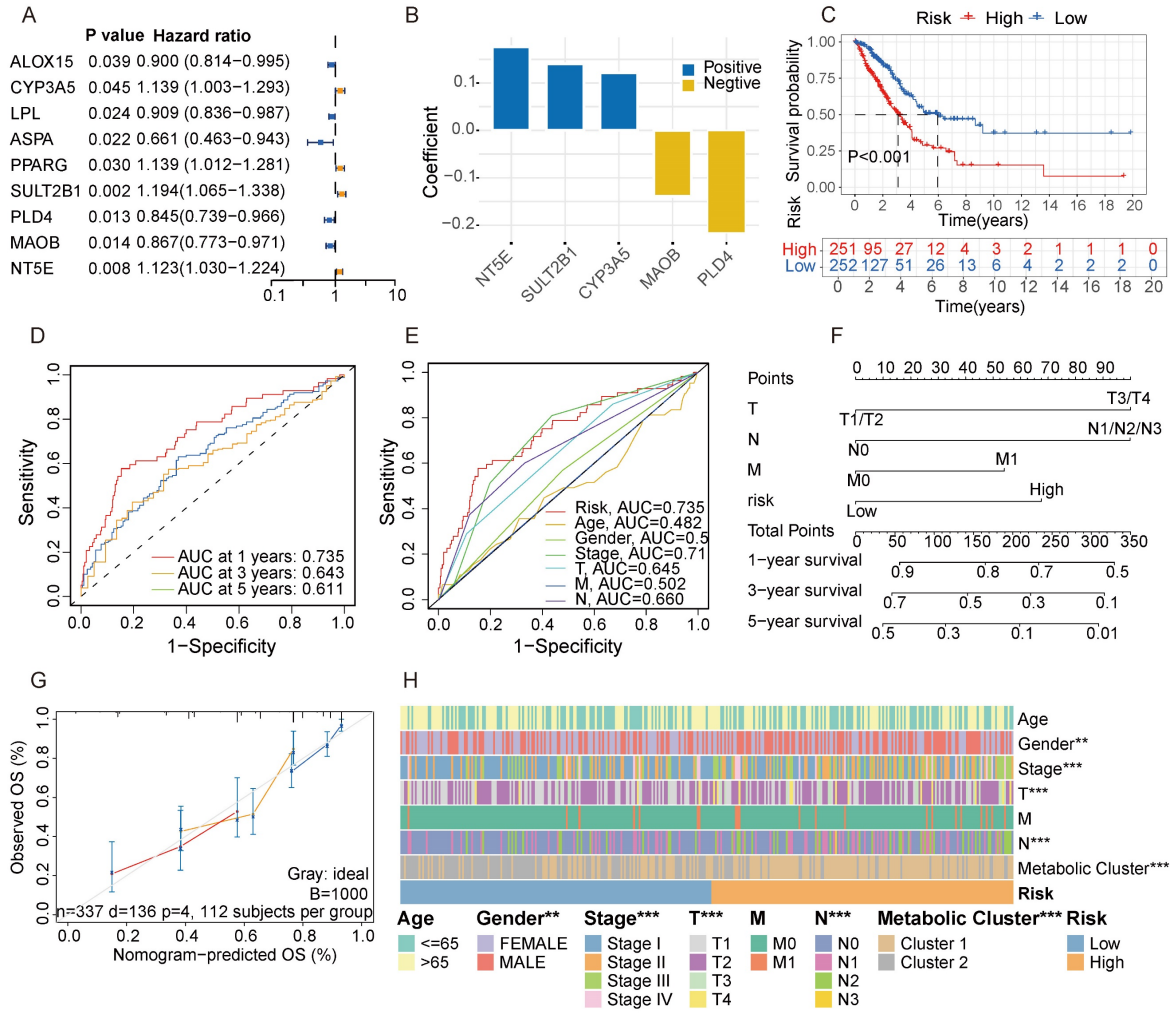
** Screening for metabolic genes with prognostic value. A.** Among the metabolite-associated gene signature, 9 genes were confirmed as independent prognostic factors through univariate analysis. **B.** The coefficient of a 5-gene risk signature identified through stepwise Cox regression analysis. **C.** Based on the expression of the 5-gene signature, patients were divided into 2 subgroups, revealing significant distinction in prognosis between high and low-risk subgroups. **D, E.** The area under the curves (AUCs) of 5-gene risk signature were significantly higher than the AUCs of age, gender, and stage. **F.** Based on LUAD data from the TCGA cohort, a nomogram was constructed incorporating independent prognostic factors (T, N, M stages) and the 5-gene risk signature. **G.** The calibration curves demonstrated favorable accuracy of the nomogram. **H.** A Heatmap demonstrated the distribution of clinical features and metabolic clusters in high and low-risk groups.

To further identify key genes in GGO, we screened for metabolic genes with prognostic value within the 30-gene signature. Among the signature, 9 genes were confirmed as independent prognostic factors through univariate analysis (Figure 4A). We then subjected these 9 genes to multivariate Cox regression analysis. According to stepwise Cox regression, we ultimately identified 5 genes as a metabolite- associated risk signature in the Cox proportional hazard model. The 5 risk genes are NT5E, SULT2B1, CYP3A5, MAOB and PLD4 (Figure 4B). The corresponding parameters of the 5-gene signature in Cox regression model was displayed in Table S2. Subsequently, we calculated risk scores for each sample based on the expression of the 5-gene signature. LUAD patients deriving from TCGA were divided into high and low-risk groups according to the median of risk score, and patients in the high-risk group had a worse prognosis with a P-value less than 0.001 (Figure 4C). The AUC of 5-gene risk signature at 1-, 3- and 5-year is 0.735, 0.643 and 0.611, respectively (Figure 4D), significantly higher than the AUCs of age (0.482), gender (0.546), and stage (0.710) (Figure 4E). We selected independent prognostic factors (T, N, M stages) and the 5-gene risk signature to draw a nomogram based on 337 LUAD patients who had complete clinical information in TCGA (Figure 4F). The calibration curves demonstrated favorable accuracy of the nomogram (Figure 4G). We also assessed the distribution of clinical features and metabolic subgroups in different risk group and demonstrated female, individuals with a lower stage and those belonging to cluster 2 were more concentrated in the low-risk group (Figure 4H).

### Sc-RNA sequencing and clinical verification identify key metabolic gene in malignant GGO

**Figure 5:**
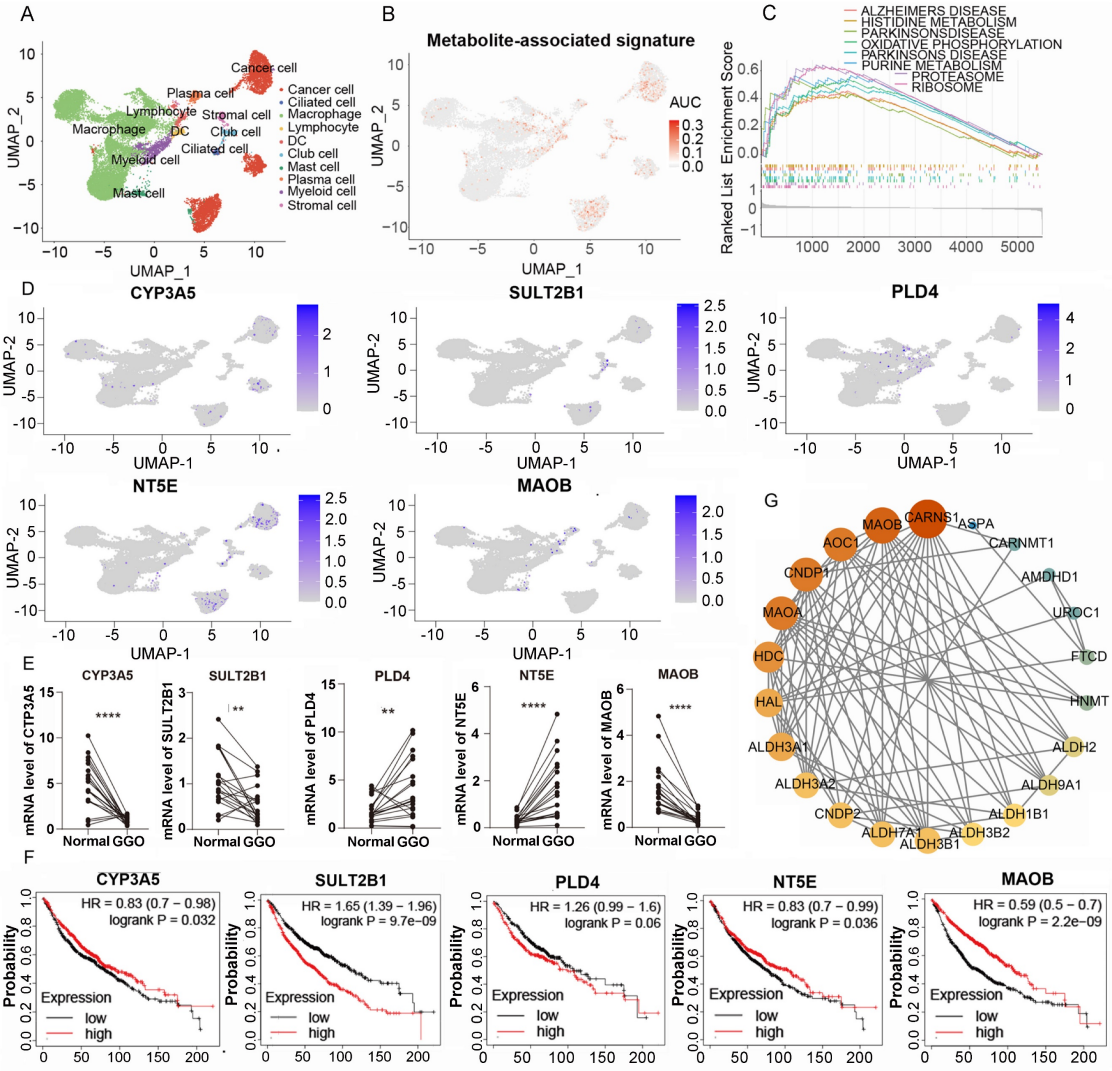
**Single-cell RNA (Sc-RNA) sequencing analysis and clinical verification identified key metabolic genes in GGO. A.** The annotated cell types of GGO tissue in GSE203360 based on uniform manifold approximation and projection analysis (UMAP). **B.** The AUC score of the 5-gene signature in Sc-RNA sequencing. **C.** Gene Set Enrichment Analysis (GSEA) showed the enriched KEGG pathways of cells exhibiting high 5-gene signature. **D.** The AUC of each gene of the 5-gene signature in Sc-RNA sequencing. **E.** RT-qPCR showed mRNA levels of 5-gene signature in 15 paired lung cancer tissues. **F.** The Kaplan-Meier plots of the 5-gene signature. **G.** Protein-protein interaction (PPI) networks of genes in histidine metabolism, suggesting the central location of MAOB.

To access the expression of metabolite-associated gene signature at the cell level, we analyzed 19391 cells derived from 5 GGO associated LUAD samples in GSE203360. Utilizing UMAP clustering based on cell-type-annotation markers (Table S1), GGO-associated cells were classified into ten cell types (Figure 5A). The AUCell algorithm identified 931 cells with high 5-gene signature, with a threshold AUC value greater than 0.034. Interestingly, cells with a high 5-gene signature were predominantly concentrated in cancer cells, rather than other cells in the tumor microenvironment (Figure 5B). Then we performed the GESA algorithm and found that the 5-gene signature-enriched cells were positively related to histidine metabolism and purine metabolism, consistent with the result of integrative omics analysis (Figure 5C). We also examined the distribution of each gene in the 5-gene signature in GGO cells and discovered PLD4, NT5E and MAOB were differentially expressed between cancer cells and non-tumor cells (Figure 5D), suggesting a more critical physiological significance. Subsequently, we collected 20 pairs of malignant GGO samples and adjacent normal tissue from pulmonary resection surgery, the mRNA level of the 5-gene signature was detected using RT-qPCR. The results of RT-qPCR suggested GGO expressed higher levels of PLD4 and NT5E, and lower levels of CYP3A5, SULT2B1 and MAOB than adjacent normal tissue (Figure 5E). Meanwhile, we generated KM plots based on prognostic data from Kaplan-Meier Plotter (Figure 5F) and only CYP3A5 and MAOB showed consistent pattern of gene expression and prognosis, which genes with lower expression in lung cancer were correlated with a better prognosis. Integrating Sc-RNA sequencing, RT-qPCR and KM plots, we identified MAOB as a key gene of GGO-associated lung cancer. Patients with lower expression level of MAOB corresponded to a better prognosis, and even in tumor microenvironment, cells expressing MAOB were mainly enriched in non-tumor cells.

The protein of MAOB catalyzes the oxidative deamination of biogenic and xenobiotic amines, and plays an important role in histidine metabolism. The protein-protein interaction (PPI) networks of genes in histidine metabolism suggesting the central location of MAOB (Figure 5G). Coincidently, the alteration of histidine metabolism and MAOB was observed in metabolomics, integrated multi-omics analysis and Sc-RNA sequencing respectively, suggesting the critical role in the pathogenesis of malignant GGO.

### MAOB suppresses proliferation, migration and invasion of lung cancer

**Figure 6:**
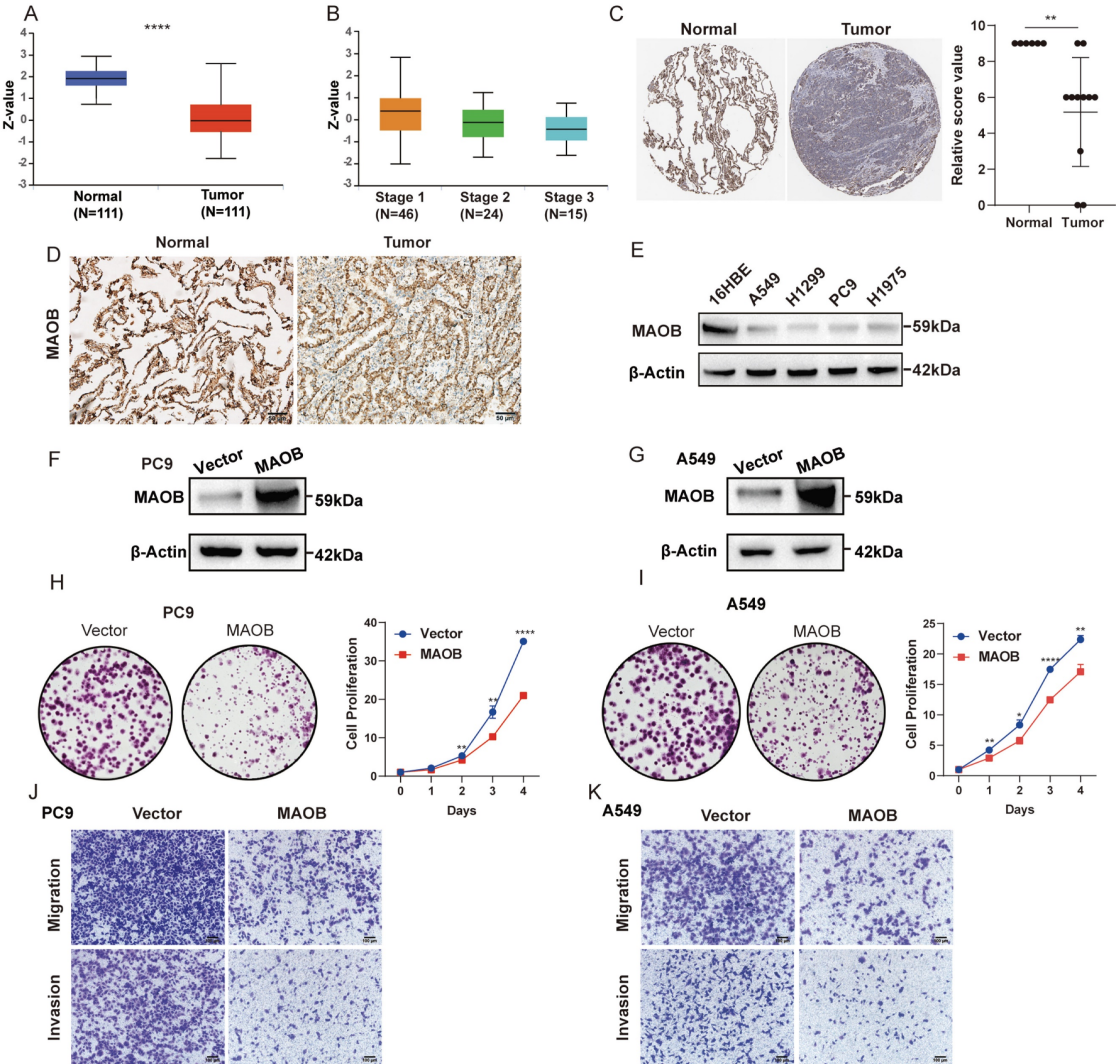
** MAOB suppresses proliferation, migration and invasion of lung adenocarcinoma in vitro. A.** The protein expression of MAOB in Clinical Proteomic Tumor Analysis Consortium (CPTAC). **B.** The protein expression of MAOB in different clinical stages. **C.** The immunohistochemistry staining of MAOB in The Human Protein Atlas (HPA). **D.** The immunohistochemistry staining of MAOB in GGO samples.** E.** Western blot detected the protein expression of MAOB in lung cancer cell lines. **F, G.** Overexpression of MAOB in PC9 and A549 cell lines. **H, I.** Overexpression of MAOB suppressed proliferation of PC9 and A549 cells in vitro. **J, K.** Overexpression of MAOB suppressed migration and invasion of PC9 and A549 cells in vitro.

Consistent with the mRNA expression pattern, we observed that LUAD tissue expressed a lower quantity of MAOB protein using the Clinical Proteomic Tumor Analysis Consortium (CPTAC) database (Figure 6A). Moreover, the protein expression decreased with the increase of clinical stage (Figure 6B). Immunohistochemical (IHC) assays derived from the Human Protein Atlas (HPA) and clinical GGO samples showed that the protein expression of MAOB was obviously reduced in lung cancer (Figure 6C and D). Western blot assays further demonstrated lung cancer cell lines expressed a lower protein level of MAOB than normal bronchial epithelial cells (Figure 6E).

**Figure 7:**
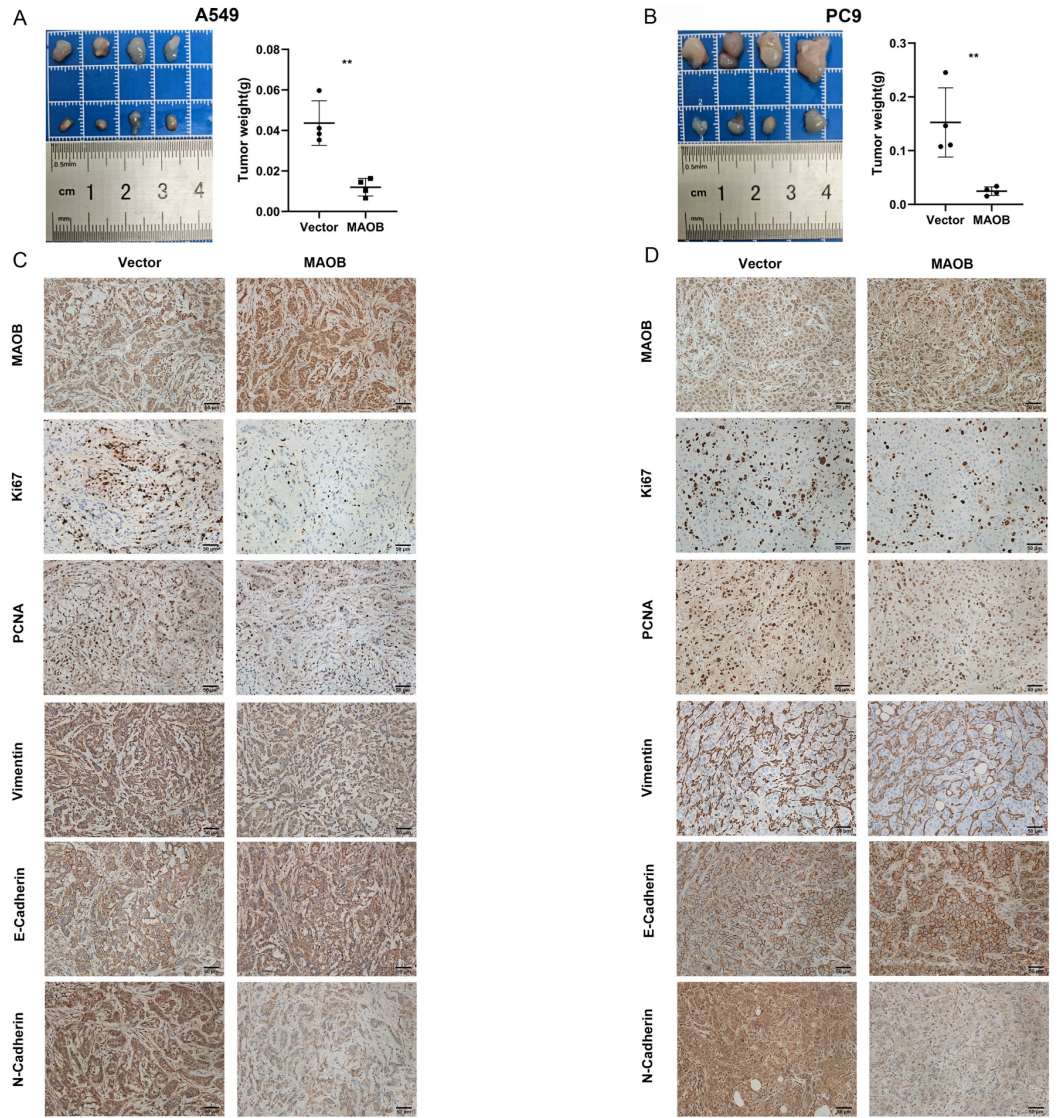
** MAOB suppresses proliferation, migration and invasion of lung adenocarcinoma in vivo. A.** MAOB suppresses the growth of tumors in nude mice. **B.** MAOB suppressed the expression of markers associated with proliferation and Epithelial-Mesenchymal Transition in tumor tissues from nude mice.

To further explore the biologic effect of MAOB on tumor progression, we overexpressed MAOB in PC9 and A549 cell lines using lentivirus. Western blot assays verified the efficiency of cell models (Figure 6F and G). Colony formation and CCK-8 assays demonstrated MAOB suppressed the proliferation of PC9 and A549 cells (Figure 6H and I). Transwell assays suggested MAOB suppressed the migration and invasion abilities of PC9 and A549 cells (Figure 6J and K). To detect the impact of MAOB on the proliferation of lung cancer in vivo, we constructed a xenograft model on nude mice. The results demonstrated MAOB suppressed the proliferation of PC9 and A549 cells in vivo (Figure 7A and B). Moreover, we conducted IHC staining for proteins associated with proliferation and Epithelial-Mesenchymal Transition (EMT) in tumor tissues from nude mice (Figure 7C and D). For proliferation-related markers, MAOB downregulated the expression of Ki67 and PCNA. For EMT-related markers, MAOB exhibited no impact on Vimentin expression but enhanced E-Cadherin expression and suppressed N-Cadherin expression. These findings imply the potential role of MAOB in influencing the proliferation and EMT progression in LUAD. Collectively, these results indicate that MAOB inhibits the proliferation, migration, and invasion of LUAD.

## Discussion

With the widespread use of CT scan, the incidence of GGO-associated lung cancer has significantly increased, especially in East Asian populations 3, 18. In comparison to solid nodules, GGO-associated lung cancer is considered to exhibit a lower malignant nature 19, preferring lepidic growth and suggesting a better prognosis 4, 5, 20. At the molecular level, GGO represents an early state of carcinogenesis, characterized by a lower tumor mutational burden and a less active immune microenvironment 7, potentially explaining the indolent clinical course. Multi-omics integrative analysis, which aims to process, compare and analyze batch data at different biomolecular levels such as genome, transcriptome, proteome and metabolome, make it possible to explore tumorigenic mechanisms from multiple dimensions. However, the limited number of studies on GGO that integrate multi-omics analysis has not yet generated disrupted metabolic networks in GGO tissue or identified target metabolic gene for tumorigenesis in GGO-associated lung cancer. Therefore, we employed a systems biology approach, integrating metabolomics, transcriptomics and Sc-RNA sequencing, to unveil disrupted metabolic pathways, a metabolite-associated gene signature and potential key gene in the carcinogenesis of GGO-associated lung cancer.

Metabolic reprogramming plays a critical role in the initiation and progression of tumors. On the one hand, tumor cells actively alter intracellular metabolic flux to meet the increased synthetic and energetic demand, exemplified by the Warburg effect 21. On the other hand, the aberrant accumulation of metabolites can directly promote tumorigenesis 22. Several metabolomics-based studies have confirmed the presence of abnormal metabolites and metabolic pathways in lung cancer, such as glycolysis and lipid metabolism 13, 23-25. However, most of these studies have focused on liquid detection (e.g., serum, urine), aiming to discover advanced metabolic biomarkers for tumor diagnosis. The few metabolomics studies using lung cancer tissue as a specimen have neither been combined with other omics to explore the genetic mechanism of tumor phenotype, nor focused on GGO-associated lung cancer. In this work, Metabolomics and transcriptomics analysis of GGO-associated lung cancer revealed 35 differential expressed metabolites and 30 metabolite-associated genes. The integrative multi-omics analysis suggested aberrant metabolic pathways in GGO tissue including fatty acid biosynthesis, purine metabolism and histidine metabolism, indicating the potential biological impact of altered metabolic pathways on genesis of lung cancer. Notably, the 30-gene signature deriving from multi-omics analysis also demonstrated prognostic and physiological significance in the TCGA cohort. LUAD patients in the TCGA cohort were classified into two metabolic clusters based on the expression of 30-gene signature. Patients in cluster 2 exhibited higher expression of multiple oncogenic metabolism and signal transduction pathways but had a better prognosis. This result aligns with a theory that metabolic reprogramming altered from premalignant lesions to invasive LUAD 26, further emphasizing the importance of the GGO metabolic landscape in understanding the carcinogenesis of lung cancer.

GGO-associated lung cancer reflects an initial status of tumor and exhibits molecular difference compared to solid nodules, commonly seen in invasive lesions. These distinctions include lower oncogenic mutation, less active immune infiltration, and a different metabolic fingerprint in GGO tissue 7, 26, 27. From this perspective, the consistent metabolic alteration observed in both GGO and solid lung cancer could be of greater significance, such alterations may unveil the mechanisms underlying tumorigenesis and progression, potentially targeted vulnerability. To explore these consistent alterations, the 30 metabolites-associated genes were further screened using univariate analysis and Cox proportional hazard model based on TCGA database. Finally, we identified 5 genes that not only differentially expressed in the GGO tissue, but also had prognostic value in the TCGA cohort which included solid nodules. At the single-cell level, Sc-RNA sequencing of a GGO cohort revealed the cells with high expression of these 5 genes were mainly concentrated in tumor cells. Several of these 5 genes have demonstrated roles in multiple solid tumors. For instance, CD73, encoded by NT5E, catalyzes the hydrolysis of extracellular AMP into adenosine. The hydrolysate promotes tumor progression by inhibiting T cell function and sustaining cancer-stem-cell traits 28-30. The protein encoded by SULT2B1 is responsible for the sulfation of cholesterol, and cholesterol sulfate synthesized by SULT2B1 in tumor cells could suppress activity of CD8 + T cells and promote glycolytic metabolism 31, 32. These findings suggested the 5 metabolite-associated genes could not only serve as potential biomarkers for predicting survival but also reveal the consistent molecular changes between initial and invasive state of lung cancer.

Multiple metabolites and enzymes associated with histidine have proven key roles in tumorigenesis. For instance, histamine, the decarboxylated product of histidine, regulates various biological processes involved in tumor progression, including angiogenesis, proliferation and immune inhibition 33, 34. The observation that histamine antagonists enhanced the efficacy of immunotherapy suggests the potential of small molecule inhibitors of histidine metabolism in cancer therapy 35. Notably, pathway enrichment analysis demonstrated histidine metabolism differently expressed at the level of metabolomics, integrative multi-omics and single-cell analyses, underscoring the significance of histidine metabolism in GGO associated lung cancer. Subsequently, we conducted a PPI network analysis and identified MAOB as a pivotal player in histidine metabolism. Consistent with the corresponding metabolic pathway, MAOB-related molecule exhibited uniform expression patterns across metabolomics, transcriptomics, Sc-RNA sequencing and clinical tissue, acting as a lower level of metabolic substrate, lower mRNA expression of single cell and clinical specimen, and better prognosis in GGO-associated lung cancer. MAOs belong to the flavin monoamine oxidase family, catalyzing oxidative deamination of amines, such as exogenous amines and neurotransmitters 36. In comparison to its isoform MAO-A, MAO-B differs in substrate affinity, tissue distribution and inhibitor specificity 37. While classically associated with Parkinson disease's vulnerability 38, 39, MAO-B has also been found to be expressed differentially between various tumors and normal tissue 40, 41. Additionally, neurotransmitters, the substrates of MAOs, have been implicated in the pathogenesis of malignancies 42. These findings suggested the potential value of MAOB in tumorigenesis, progression and metastasis. In this work, we detected the mRNA and protein expression in clinical specimen and LUAD cell lines, and explored the role of MAOB in tumor proliferation and metastasis in vitro and vivo. The results demonstrated MAOB was lower expressed in clinical specimen and cell lines, and overexpression of MAOB suppressed the proliferation and metastasis of LUAD in vitro and vivo. These findings highlight MAO-B could be a potential vulnerability of LUAD therapy.

In conclusion, this study represents the first comprehensive utilizing metabolomics, transcriptomics, and Sc-RNA sequencing, to identify unique metabolite-associated gene signature, disrupted histidine metabolism, and pinpoint MAOB as a key gene in GGO-associated lung cancer. The functional experiments further revealed MAOB suppressed the proliferation and metastasis of LUAD in vitro and vivo. These findings contribute valuable insights into the molecular landscape of GGO-associated lung cancer and highlight MAOB as a promising target for therapeutic intervention in lung cancer.

## Supplementary Material

Supplementary figures and tables.

## Figures and Tables

**Table 1:** Primers set for RT-qPCR analysis of the 5-gene signature

Primer	Primer sequence
CYP3A5-F	ACCTACCTATGATGCCGTGG
CYP3A5-R	TTTGGGTCATGGTGAAGAGC
SULT2B1-F	TGAGCTCCCATCTTCCCATC
SULT2B1-R	GAAGCCAGCCCTTAATGTGG
PLD4-F	TCACTTCAACCGTTTCCAGC
PLD4-R	ATAGATGAACTCCTGGGCGC
NT5E-F	GCTCTTCACCAAGGTTCAGC
NT5E-R	TCGATCAGTCCTTCCACACC
MAOB-F	AGAAGAAGCTCCAGTTGCCT
MAOB-R	TGCTCCTCACACCAGTTCTT

**Table 2:** Clinical and pathological characteristics of study cohort

Characteristics	Total	Metabolomics	Transcriptomics	P
Number	40	15	25	
Age (median±SD)	52.0 ± 9.2	51.6 ± 7.0	52.2 ± 10.5	0.86
Gender (%)				0.85
Male	10 (25)	4 (26.7)	6 (24.0)	
Female	30 (75)	11 (73.3)	19 (76.0)	
Smoking status (%)				0.28
Ex-smoker	31 (77.5)	13 (86.7)	18 (72.0)	
Non-smoker	9 (22.5)	2 (13.3)	7 (28.0)	
Localization (%)				0.39
Right	26 (65.0)	11 (73.3)	15 (60.0)	
Left	14 (35.0)	4 (26.7)	10 (40.0)	
Size (median [IQR])	8.3 [7.3,10.0]	9.0 [8.0,10.0]	8.0 [7.0,9.8]	0.07
Component type (%)				0.18
Pure GGO	24 (60.0)	11 (73.3)	13 (52.0)	
Mixed GGO	16 (40.0)	4 (26.7)	12 (48.0)	
Pathology type				0.46
In situ	10 (25.0)	4 (26.7)	6 (24.0)	
Minimally invasive	12 (30.0)	6 (40.0)	6 (24.0)	
Invasive	18 (45.0)	5 (33.3)	13 (52.0)	

*SD* standard deviation, *IQR* interquartile range, *GGO* ground-glass opacity

## Abbreviations

GGO: ground-glass opacity
LUAD: lung adenocarcinoma
CT: computed tomography
ULCAP: early lung cancer action program
OPLS-DA: orthogonal partial least squares-discriminant analysis
GC-TOF/MS: time-of-flight mass spectrometer with gas chromatography
DEM: differentially expressed metabolite
VIP: variable importance in projection
SAM: Significance analysis of microarray
FDR: false discovery rate
FC: fold change
DEG: differentially expressed gene
PCA: Principal component analysis
MAG: metabolites-associated gene
KEGG: Kyoto Encyclopedia of Genes and Genomes
TCGA: Cancer Genome Atlas Program
AIC: Akaike information criterion
ROC: receiver operating characteristic
UAMP: uniform manifold approximation and projection analysis
RT-qPCR: quantitative real-time polymerase chain reaction
FBS: fetal bovine serum
IHC: immunohistochemistry
CPTAC: Clinical Proteomic Tumor Analysis Consortium
HPA: Human Protein Atlas

## References

[1] Chung M, Tam K, Wallace C, Yip R, Yankelevitz DF, Henschke CI (2017). International Early Lung Cancer Action Program: update on lung cancer screening and the management of CT screen-detected findings. AME Medical Journal.

[2] Henschke CI, Yankelevitz DF, Mirtcheva R, McGuinness G, McCauley D, Miettinen OS (2002). CT screening for lung cancer: frequency and significance of part-solid and nonsolid nodules. American Journal of Roentgenology.

[3] Zhang Y, Jheon S, Li H, Zhang H, Xie Y, Qian B (2020). Results of low-dose computed tomography as a regular health examination among Chinese hospital employees. The Journal of thoracic and cardiovascular surgery.

[4] Hattori A, Hirayama S, Matsunaga T, Hayashi T, Takamochi K, Oh S (2019). Distinct clinicopathologic characteristics and prognosis based on the presence of ground glass opacity component in clinical stage IA lung adenocarcinoma. J Thorac Oncol.

[5] Berry MF, Gao R, Kunder CA, Backhus L, Khuong A, Kadoch M (2018). Presence of even a small ground-glass component in lung adenocarcinoma predicts better survival. Clin Lung Cancer.

[6] Aokage K, Suzuki K, Saji H, Wakabayashi M, Kataoka T, Sekino Y (2023). Segmentectomy for ground-glass-dominant lung cancer with a tumour diameter of 3 cm or less including ground-glass opacity (JCOG1211): a multicentre, single-arm, confirmatory, phase 3 trial. The Lancet Respiratory Medicine.

[7] Chen K, Bai J, Reuben A, Zhao H, Kang G, Zhang C (2021). Multiomics analysis reveals distinct immunogenomic features of lung cancer with ground-glass opacity. Am J Respir Crit Care Med.

[8] Kobayashi Y, Mitsudomi T, Sakao Y, Yatabe Y (2015). Genetic features of pulmonary adenocarcinoma presenting with ground-glass nodules: the differences between nodules with and without growth. Ann Oncol.

[9] Zhang C, Zhang J, Xu F-P, Wang Y-G, Xie Z, Su J (2019). Genomic landscape and immune microenvironment features of preinvasive and early invasive lung adenocarcinoma. J Thorac Oncol.

[10] Pavlova NN, Zhu J, Thompson CB (2022). The hallmarks of cancer metabolism: Still emerging. Cell Metab.

[11] Finley LW (2023). What is cancer metabolism?. Cell.

[12] Xu J-Q, Fu Y-L, Zhang J, Zhang K-Y, Ma J, Tang J-Y (2022). Targeting glycolysis in non-small cell lung cancer: Promises and challenges. Front Pharmacol.

[13] Wang G, Qiu M, Xing X, Zhou J, Yao H, Li M (2022). Lung cancer scRNA-seq and lipidomics reveal aberrant lipid metabolism for early-stage diagnosis. Sci Transl Med.

[14] Mathé EA, Patterson AD, Haznadar M, Manna SK, Krausz KW, Bowman ED (2014). Noninvasive urinary metabolomic profiling identifies diagnostic and prognostic markers in lung cancer. Cancer Res.

[15] Thaiparambil J, Dong J, Grimm SL, Perera D, Ambati CSR, Putluri V (2023). Integrative metabolomics and transcriptomics analysis reveals novel therapeutic vulnerabilities in lung cancer. Cancer Medicine.

[16] Collins J, Stern EJ (1997). Ground-glass opacity at CT: the ABCs. AJR American journal of roentgenology.

[17] Akaike H (1974). A new look at the statistical model identification. IEEE transactions on automatic control.

[18] Yankelevitz DF, Yip R, Smith JP, Liang M, Liu Y, Xu DM (2015). CT screening for lung cancer: nonsolid nodules in baseline and annual repeat rounds. Radiology.

[19] Travis WD, Asamura H, Bankier AA, Beasley MB, Detterbeck F, Flieder DB (2016). The IASLC lung cancer staging project: proposals for coding T categories for subsolid nodules and assessment of tumor size in part-solid tumors in the forthcoming eighth edition of the TNM classification of lung cancer. J Thorac Oncol.

[20] Hattori A, Matsunaga T, Takamochi K, Oh S, Suzuki K (2017). Importance of ground glass opacity component in clinical stage IA radiologic invasive lung cancer. The Annals of Thoracic Surgery.

[21] DeBerardinis RJ, Chandel NS (2020). We need to talk about the Warburg effect. Nature metabolism.

[22] Thompson CB (2009). Metabolic enzymes as oncogenes or tumor suppressors. The New England journal of medicine.

[23] Huang L, Wang L, Hu X, Chen S, Tao Y, Su H (2020). Machine learning of serum metabolic patterns encodes early-stage lung adenocarcinoma. Nature communications.

[24] Seow WJ, Shu X-O, Nicholson JK, Holmes E, Walker DI, Hu W (2019). Association of untargeted urinary metabolomics and lung cancer risk among never-smoking women in China. JAMA Network Open.

[25] Yu L, Li K, Zhang X (2017). Next-generation metabolomics in lung cancer diagnosis, treatment and precision medicine: mini review. Oncotarget.

[26] Nie M, Yao K, Zhu X, Chen N, Xiao N, Wang Y (2021). Evolutionary metabolic landscape from preneoplasia to invasive lung adenocarcinoma. Nature communications.

[27] Shang J, Jiang H, Zhao Y, Lai J, Shi L, Yang J (2023). Differences of molecular events driving pathological and radiological progression of lung adenocarcinoma. EBioMedicine.

[28] Ma X-L, Hu B, Tang W-G, Xie S-H, Ren N, Guo L (2020). CD73 sustained cancer-stem-cell traits by promoting SOX9 expression and stability in hepatocellular carcinoma. J Hematol Oncol.

[29] Le X, Negrao MV, Reuben A, Federico L, Diao L, McGrail D (2021). Characterization of the immune landscape of EGFR-mutant NSCLC identifies CD73/adenosine pathway as a potential therapeutic target. J Thorac Oncol.

[30] King RJ, Shukla SK, He C, Vernucci E, Thakur R, Attri KS (2022). CD73 induces GM-CSF/MDSC-mediated suppression of T cells to accelerate pancreatic cancer pathogenesis. Oncogene.

[31] Zhao T, Li Y, Shen K, Wang Q, Zhang J (2021). Knockdown of OLR1 weakens glycolytic metabolism to repress colon cancer cell proliferation and chemoresistance by downregulating SULT2B1 via c-MYC. Cell Death Dis.

[32] Wang S, Wang R, Xu N, Wei X, Yang Y, Lian Z SULT2B1-CS-DOCK2 axis regulates effector T cell exhaustion in hepatocellular carcinoma microenvironment. Hepatology. 2023: 10.1097.

[33] Chen J, Liu G, Wang X, Hong H, Li T, Li L (2022). Glioblastoma stem cell-specific histamine secretion drives pro-angiogenic tumor microenvironment remodeling. Cell Stem Cell.

[34] Moya-García AA, Pino-Ángeles A, Sánchez-Jiménez F, Urdiales JL, Medina MÁ (2021). Histamine, metabolic remodelling and angiogenesis: a systems level approach. Biomolecules.

[35] Liu P, Kroemer G, Kepp O (2023). Histamine antagonists promote cancer immunosurveillance. Oncoimmunology.

[36] Sturza A, Leisegang MS, Babelova A, Schröder K, Benkhoff S, Loot AE (2013). Monoamine oxidases are mediators of endothelial dysfunction in the mouse aorta. Hypertension.

[37] Tipton KF (2018). 90 years of monoamine oxidase: some progress and some confusion. J Neural Transm.

[38] Gray R, Patel S, Ives N, Rick C, Woolley R, Muzerengi S (2022). Long-term effectiveness of adjuvant treatment with catechol-O-methyltransferase or monoamine oxidase B inhibitors compared with dopamine agonists among patients with Parkinson disease uncontrolled by levodopa therapy: the PD MED randomized clinical trial. JAMA neurology.

[39] Wu Z, Xia Y, Wang Z, Su Kang S, Lei K, Liu X (2021). C/EBPβ/δ-secretase signaling mediates Parkinson's disease pathogenesis via regulating transcription and proteolytic cleavage of α-synuclein and MAOB. Mol Psychiatry.

[40] Zhang Z, Ma Y, Guo X, Du Y, Zhu Q, Wang X (2021). FDX1 can impact the prognosis and mediate the metabolism of lung adenocarcinoma. Front Pharmacol.

[41] Xu N, Wu Y-P, Ke Z-B, Liang Y-C, Cai H, Su W-T (2019). Identification of key DNA methylation-driven genes in prostate adenocarcinoma: an integrative analysis of TCGA methylation data. J Transl Med.

[42] Zahalka AH, Frenette PS (2020). Nerves in cancer. Nature Reviews Cancer.

