# Supplementary material for: Integrative Omics Analysis Reveals Metabolic Features of Ground-Glass Opacity-Associated Lung Cancer
Source: J Cancer. 2024 Feb 4;15(7):1848–62. doi: 10.7150/jca.92437 (PMC10905408; doi:10.7150/jca.92437)
Supplement: Supplementary file 1 — Supplementary figures and tables. [file jcav15p1848s1.pdf]

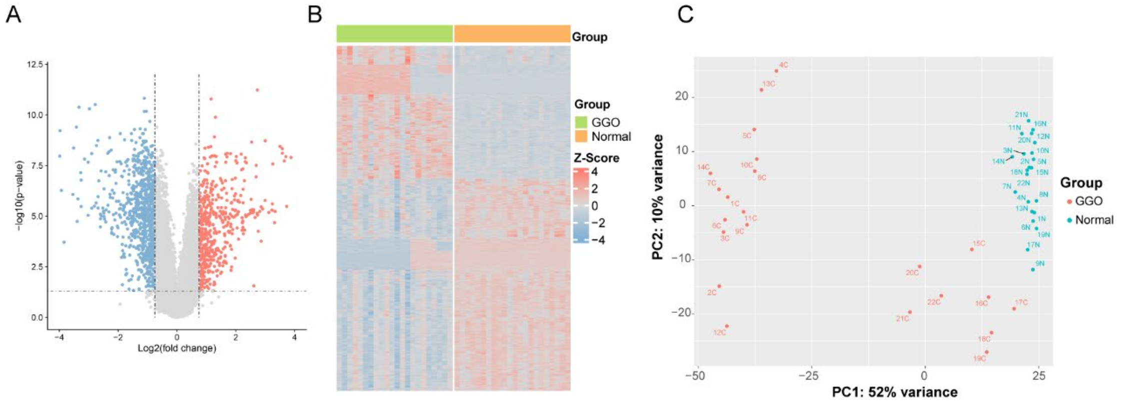

Figure S1. Transcriptomics analysis of GGO-associated lung cancer and adjacent normal tissue. **A.** The volcano plot illustrated differentially expressed genes (DEGs) between GGO and adjacent normal tissue (P<0.01, $\log_2$(FC) exceeding 0.75X). **B.** The heatmap visualized the expression patterns of DEGs between GGO and adjacent normal tissue. C. Principal Component Analysis (PCA) analysis demonstrated a clear separation between samples from different groups.

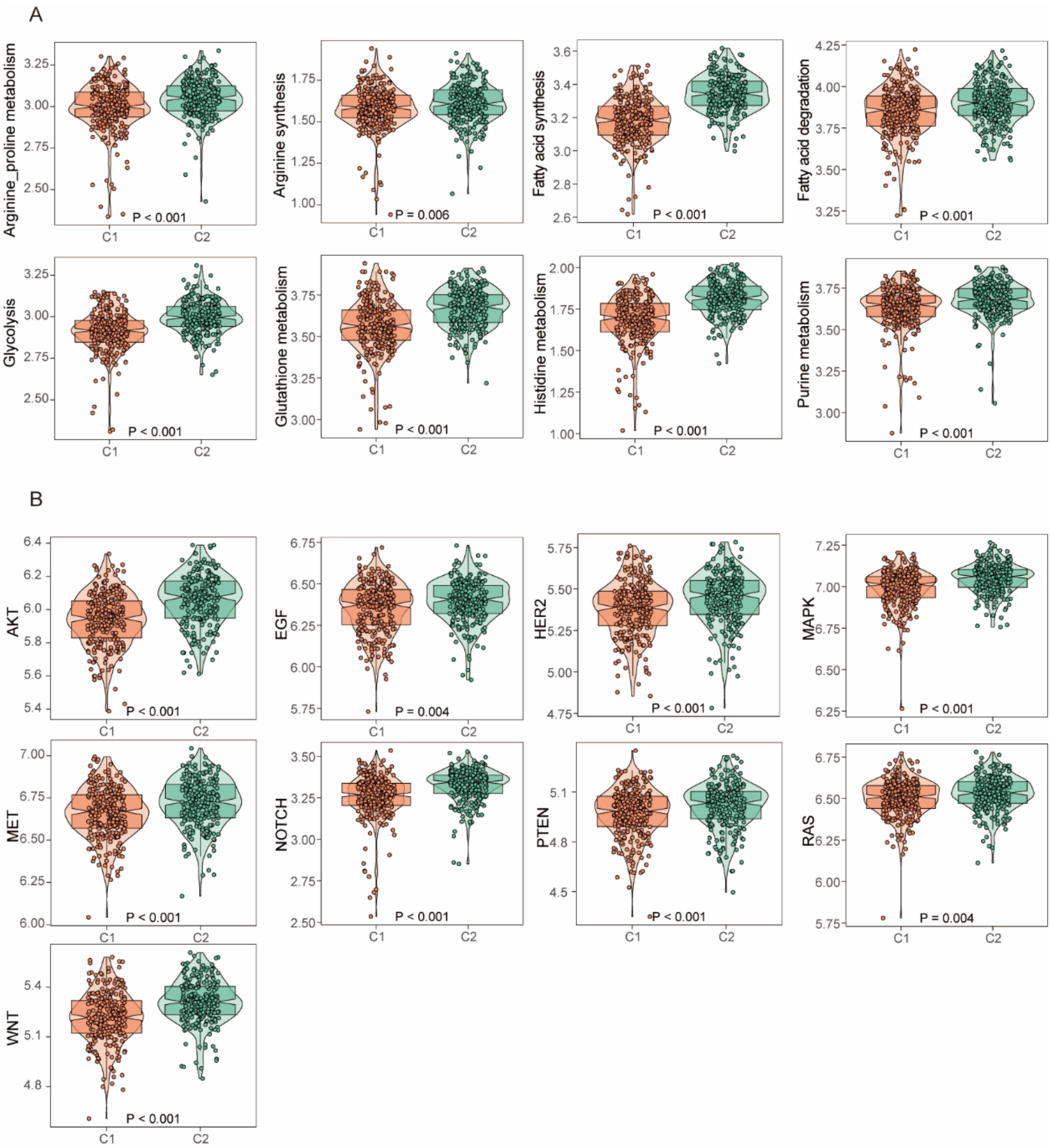

Figure S2. Patients with different metabolic clusters exhibited distinct molecular features. **A.** Expression of tumor-associated metabolic pathways in patients with different metabolic clusters. **B.** Expression of tumor-associated physiological pathways in patients with different metabolic clusters.

Table S1 Cell-type-annotation markers for GGO-associated cells

Cell type	Marker
Macrophages	LYZ, CD68, MRC1, C1OA
Club cells	SCGB1A1, SCGB3A1, SCGB3A2, EPCAM
Myeloid cells	MKI67, TOP2A, LYZ
Dendritic cells	CDC, FCER1A, CLEC10A
T cells	CD2, TRBC2, CD3D
Plasma cells	CD79A, JCHAIN, MZB1, IGKC
Mast cells	TPSAB1, TPSAB2, CPA3
Stromal cells	CDH5, CLDN5VW, DCN, LUM, COL1A2
Ciliated cells	TPPP3, PIFO, EPCAM
Cancer cells	SFTPB, SFTPC, EPCAM, NAPSA, NKX2-1

Table S2 The parameters of 5-gene signature in Cox regression model

Gene	Coefficient	Hazard ratio	P-value
CYP3A5	0.122	1.129 (0.988-1.291)	0.075
SULT2B1	0.141	1.151 (1.030-1.286)	0.013
PLD4	-0.216	0.806 (0.693-0.937)	0.005
MAOB	-0.139	0.870 (0.766-0.988)	0.031
NT5E	0.176	1.193 (1.085-1.312)	<0.001
